# Impact of hyaluronic acid injection on the knee joint friction

**DOI:** 10.1007/s00167-023-07602-w

**Published:** 2023-10-16

**Authors:** Luisa de Roy, Kerstin Eichhorn, Martin Faschingbauer, Klaus Schlickenrieder, Anita Ignatius, Andreas Martin Seitz

**Affiliations:** 1 Institute of Orthopedic Research and Biomechanics, Center for Trauma Research, Ulm University Medical Center, Helmholtzstraße 14, 89081 Ulm, Germany; 2 Department of Orthopedic Surgery, RKU, Ulm University Medical Center, Ulm, Germany; 3 Faculty of Production Engineering and Management, Ulm University of Applied Sciences, Ulm, Germany

**Keywords:** Friction, Cartilage, Meniscus, Degeneration, Hyaluronic acid

## Abstract

**Purpose:**

The purpose of this in vitro study was to investigate whether or not hyaluronic acid supplementation improves knee joint friction during osteoarthritis progression under gait-like loading conditions.

**Methods:**

Twelve human cadaveric knee joints were equally divided into mild and moderate osteoarthritic groups. After initial conservative preparation, a passive pendulum setup was used to test the whole joints under gait-like conditions before and after hyaluronic acid supplementation. The friction-related damping properties given by the coefficient of friction *µ* and the damping coefficient c (in kg m^2^/s) were calculated from the decaying flexion–extension motion of the knee. Subsequently, tibial and femoral cartilage and meniscus samples were extracted from the joints and tested in an established dynamic pin-on-plate tribometer using synthetic synovial fluid followed by synthetic synovial fluid supplemented with hyaluronic acid as lubricant. Friction was quantified by calculating the coefficient of friction.

**Results:**

In the pendulum tests, the moderate OA group indicated significantly lower *c*_0_ values (*p* < 0.05) under stance phase conditions and significantly lower *µ*_0_ (*p* = 0.01) values under swing phase conditions. No degeneration-related statistical differences were found for *µ*_end_ or *c*_end_. Friction was not significantly different (*p* > 0.05) with regard to mild and moderate osteoarthritis in the pin-on-plate tests. Additionally, hyaluronic acid did not affect friction in both, the pendulum (*p* > 0.05) and pin-on-plate friction tests (*p* > 0.05).

**Conclusion:**

The results of this in vitro study suggested that the friction of cadaveric knee joint tissues does not increase with progressing degeneration. Moreover, hyaluronic acid viscosupplementation does not lead to an initial decrease in knee joint friction.

## Introduction

Despite great scientific achievements in understanding osteoarthritis in recent decades, the multifactorial etiology of knee osteoarthritis (OA) still remains a challenge for joint-preserving treatment strategies [1]. Macroscopically, the disease is characterized by progressive degradation of cartilage and meniscus tissue. From a tribological point of view, this can be attributed to alterations in joint friction, including lubrication by the synovial fluid (SF) [2–4]. In particular, research on joint lubrication is of great clinical interest, because it is the target of treatment strategies based on tribo-supplementation. These include intra-articular injections of corticosteroids, platelet-rich plasma and hyaluronic acid (HA) as the most commonly used supplements [5–7].

The rationale for supplementing HA is given by pathological changes of the SF composition during OA progression [8]. A decline in HA concentration and shift from high to lower molecular weight (MW) causes a reduced viscosity, thus impaired lubricity of the SF [8, 9]. Therefore, supplementing high MW HA aims to restore the physiologic properties of SF in terms of joint lubrication to maintain a low-friction environment [8]. Although the improvement of joint lubrication appears to be the predominant therapeutic target of HA supplementation, its mechanical impact is currently not fully understood [5, 8, 10]. Controversial literature regarding the mechanism of action of HA supplementation contribute to discrepancies in national and international treatment guidelines [1]. Thus, there is still the need for investigations on its various therapeutic effects. Previously, a variety of pendulum and pin-on-plate setups were used to investigate the tribological effect of HA supplementation at the joint [11–13] and tissue levels [14–16], respectively. In whole animal knee joints, HA was reported to have a friction-reducing effect [12, 17]. At present, comparable in vitro friction studies on whole naturally degenerated human knee joints are lacking. At the tissue level, HA reduced friction of degenerated human cartilage samples under quasi-static testing conditions [16]. However, studies on more physiologic conditions are still required. Under gait-like loading conditions the double-peak loading profile with the associated different velocities lead to the characteristic lubrication regimes, which are crucial for biotribological investigations of the knee joint tissues [17–19]. In conclusion, there is a lack of knowledge about both, the general friction properties and the tribomechanical effect of HA in the aging human knee joint. Therefore, the aim of the present study was to perform a two-part tribological study on degenerated human knee joints. On the basis of the characteristic progressive cartilage damage and the associated surface roughening [3, 20, 21], we hypothesized that joint friction is higher in moderate OA joints compared to mild OA joints. Moreover, based on the friction-minimizing effect found in experimental OA models after HA injection [14, 17], we hypothesized that HA supplementation is able to reduce friction in both, mild and moderate OA knees. Overall, this in-vitro tribological study extends the literature by providing insights into the friction properties and the tribomechanical effect of HA on degenerated human knee joint tissues under simulated gait conditions.

## Material and methods

### Study design

**Fig. 1:**
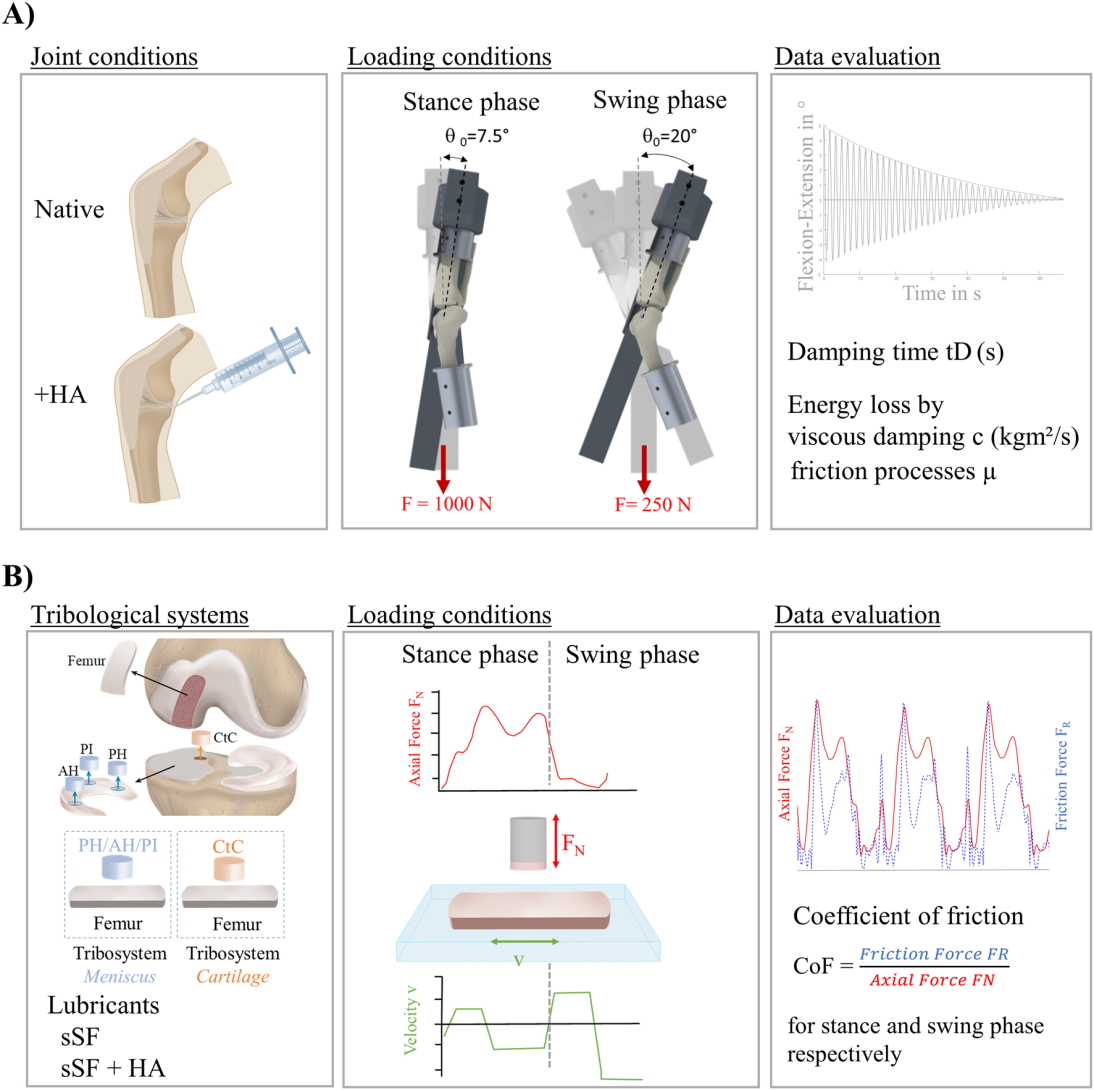
Study design overview; (**A**) Hyaluronic acid (HA) supplementation at the joint level was examined using a passive pendulum friction setup (upper row), while the load application for the stance and swing phases were derived from a human gait cycle [24]. A viscous friction model was applied on the decaying passive knee joint motion in flexion–extension to calculate the damping time (tD) and to quantify the energy loss in terms of viscous damping (c) and friction processes (µ). (**B**) Testing of HA supplementation at the tissue level where meniscus against cartilage (tribosystem Meniscus) and tibial against femoral cartilage (tribosystem Cartilage) samples were examined using a dynamic pin-on-plate tribometer under gait-like loading conditions [19], while the coefficient of friction (CoF) was separately calculated for both, the stance and swing phases

Friction tests on aged human knee joints with signs of mild or moderate OA were performed using a passive pendulum friction setup and a dynamic pin-on-plate tribometer [19, 22] to investigate whole joint friction and the tribological effect of HA supplementation at the joint level and on extracted meniscus and cartilage samples at the tissue level, respectively (Fig. 1). In both tests, the load application was performed in accordance to the swing and stance phases of gait [23, 24].

### Macroscopic assessment of OA severity

On the basis of a-priori sample size calculation using a comparable study design (G*Power 3.1.9.7 ([12], effect size *d* = 2.10, *α* error = 0.05, power (1 − *β*) = 0.95), twelve human cadaveric knee joints were obtained from an official tissue bank (Science Care, Phoenix (AZ), USA) and equally divided into a mild and a moderate OA group (exclusion criteria: prior knee surgery, cancer). On the basis of correlations between age and OA severity [25], the donor age was initially used as a selection criteria, which was ≤ 65 years for the mild OA (four females, two males, mean age: 49 ± 8 years) and > 65 years for the moderate OA joints (three females, three males, mean age: 80 ± 5 years). OA severity of the joints was macroscopically assessed using a semi-quantitative evaluation based on the whole-organ arthroscopic knee score (WOAKS) [26]. The articular cartilage was evaluated in accordance with the International Cartilage Research Society scoring system [27] at standardized locations on the femur (*n* = 21), tibia (*n* = 18) and patella (*n* = 9). Meniscus damage was analyzed based on the scoring system from Pauli et al. [28] (range 0 to 4) at standardized locations on the femoral- (*n* = 9) and tibial-facing sides (*n* = 9) of the menisci. The whole joint score was then calculated by summarizing all scores, ranging from 0 to 264.

### Passive pendulum tests

#### Sample preparation

The joints were stored at − 20 °C and thawed for 36 h at 4 °C before preparation. Subsequently, the skin, fat and muscle tissue were removed, while the collateral ligaments, the patellar ligament at the tibia and the joint capsule were maintained intact. The bony ends of the femur and tibia were embedded in custom-made bone cylinders using polymethyl-methacrylate (Technovit 3040, Heraeus Kulzer GmbH, Wertheim, Germany) for fixation of the knees in the pendulum setup [22]. Two customized bone screws were bi-cortically anchored in the femur and tibia, while coordinate systems were aligned along the anatomical axes of the bones for kinematic measurements. The joints were kept moist with isotonic saline solution throughout the preparation process.

#### Setup and testing routine

Following preparation, the knee joint was mounted vertically inverted in an inclined mounting block on a base plate of a previously introduced pendulum setup [22]. The tibia was allowed to freely oscillate relative to the femur with the pendulum arm attached. Joint loading under simulated swing phase conditions [24] was achieved by applying a dead weight of 250 N to the pendulum with an initial deflection angle of 20° [24]. For simulation of the stance phase [24], a dead weight of 1000 N was applied and the initial defection was lowered to 7.5° [24, 29]. The mounting angle between the femur and tibia was set to 160° for swing phase conditions and 172.5° for stance phase conditions to reproduce in vivo tibiofemoral contact mechanics [29]. A dead weight of 10 N was attached at the patella tendon using a wire rope [30] to increase joint stability. Passive joint motion was initiated by releasing the pendulum from the respective initial deflection. A motion capturing system (Prime 13, NaturalPoint; nine cameras, mean error after calibration < 0.3 mm, 240 fps image acquisition rate) was used to record the three-dimensional decaying motion of the oscillation. The joints were tested eight times in the native condition under both, the stance and swing phase loading conditions, resulting in a total of 16 test runs. Between the stance and swing phase conditions, the axial loading of the knee was removed for 20 min to allow for soft tissue relaxation. Following the tests in the native condition, 6 ml HA (Synvisc, Hylan G-F 20, Sanofi-Aventis, GmbH, Frankfurt, Germany, HA concentration: 8 mg/ml; MW: 6000 kDa) were injected into the joint capsule using a 22G syringe (B. Braun Melsungen AG, Germany), which corresponds to a clinical one-time treatment procedure [31]. The knees were manually flexed and extended five times to homogeneously distribute the HA in the joint. The joints were then similarly tested as described in the native condition.

Data evaluation was performed using a customized MATLAB script (MATLAB 2022a, the MathWorks Inc., Natick, United States), where a viscous friction model [32] (Eq. 1) was applied to the decaying flexion–extension data in the sagittal plane to quantify the energy loss by viscous damping (*c* in kg m/s^2^) and friction processes (*µ*).

1 $$\theta_t = \theta_0 * \left[ {e^{ - 2\zeta t/T} - \frac{\mu mgrT^2 }{{4\pi^2 I\theta_0 }}* \frac{{1 + e^{ - \zeta } }}{{1 - e^{ - \zeta } }}*\left( {1 - e^{ - 2\zeta t/T} } \right)} \right]$$

with,

2 $$\zeta = \frac{{\frac{cT}{{4L}}}}{{\sqrt {{1 - \left( {\frac{cT}{{4\pi L}}} \right)^2 }} }}$$

and *θ*_0_ being the starting deflection, *t* = time, *m* = pendulum mass, *g* = gravity, *r* = radius of the femoral condyles, *T* = periodic time and *I* = moment of inertia about the joint rotation axis [32]. To analyze the energy loss regardless of any mathematical model, the damping time (tD) of the pendulum motion was determined. The data of the first (*c*_0_, *µ*_0_ and tD_0_) and the eighth test run (*c*_end_, *µ*_end_ and tD_end_) were evaluated to analyze the influence of the time-dependent soft tissue behavior on the results.

### Pin-on-plate tests

#### Sample preparation

After the pendulum tests, the capsuloligamentous structures of the knee joint were removed, while care was taken to avoid damaging the femoral and tibial cartilage as well as the menisci. The remaining tissues were rinsed in phosphate-buffered solution (PBS; Fisher Scientific GmbH, Schwerte, Germany) to remove the supplemented HA. Subsequently, flat cartilage plates (20 × 40 mm) were extracted from the lateral femur condyles using a microtome blade as previously described [19, 33]. Furthermore, cylindrical osteochondral cores were harvested from the cartilage-to-cartilage contact area next to the eminentia intercondylaris of the lateral compartment using a trephine drill (∅ 6 mm, Geiz Dental, Leipzig, Germany). A total of three cylindrical plugs were punched out from the anterior horn, posterior horn and pars intermedia from the according lateral menisci using a biopsy punch (∅ 6 mm, Stiefel, GSK company, United Kingdom). Consequently, the following tissue parings were tested: the femoral-facing side of the meniscus against femoral cartilage (tribosystem Meniscus) and the tibial osteochondral core against the flat femoral cartilage (tribosystem Cartilage).

#### Setup and testing routine

Both tribological systems were tested under simulated gait-like loading conditions (loading profile of combined stance and swing phases) using a dynamic pin-on-plate tribometer [19]. The test order of both, the cartilage and menisci pins were randomized because all pins were tested against the same femoral cartilage plate. First, the samples were lubricated with 0.3 ml synthetic synovial fluid (sSF), with a HA concentration of 3 mg/ml and a MW of 1.3*10^6^ Da [34]. Second, 0.3 ml sSF was mixed with HA at a ratio of 1:1 (sSF + HA) [35]. During a testing time of 600 s (equals approximately 545 applied gait-like loading cycles), the axial force (*F*_N_) and friction force (*F*_R_) were recorded using a multiaxial load cell (sample rate 100 Hz, accuracy class: 0.5%, ME-Messsysteme GmbH, Henningsdorf, Germany) which was adapted to a dynamic testing machine (ElectroForce 5500, TA Instruments, New Castle, USA). The coefficient of friction (CoF) was calculated according to Coulomb’s law (Eq. 3). First, the data of the gait-like loading profile were separated into stance and swing phase of gait to be able to differentiate the CoF between stance and swing phase conditions. To analyze potential effects of the testing time on friction [19] the CoF was averaged from the first three (CoF_0_) and last three (CoF_end_) cycles, for both the stance and swing phase, respectively.

3 $${\text{CoF}} = { }\frac{F_R }{{F_N }}$$

### Statistics

All statistical analyses were performed using a statistical software package (GraphPad Prism 8.4.3, GraphPad Software Inc., Boston, USA). Normal distribution was analyzed for all evaluated parameters using Shapiro–Wilk testing. The whole-joint scores showed normal distributed data. Therefore, the joints scores of the mild and moderate OA joints were compared using unpaired t-testing. The parameters tD, *µ* and *c* of the pendulum tests and the CoF of the pin-on-plate tests indicated non-normally distributed data. Consequently, differences in tD, *µ* and *c* at the timepoints *t*_0_ and *t*_end_ were analyzed using Wilcoxon testing, which was also applied for the comparison of the tests in the native condition with those after HA supplementation. Differences between knee joints with mild and moderate OA were investigated using Mann–Whitney test. The results of the pin-on-plate friction tests were statistically analyzed using Wilcoxon testing for the comparison of the CoF_0_ and CoF_end_ and the comparison of the tests with the different lubricants (sSF against sSF + HA). Differences between the CoF of mild and moderate OA samples were evaluated using Mann–Whitney testing. *p* ≤ 0.05 was considered to be statistically significant.

## Results

### Macroscopic assessment of OA severity

**Fig. 2:**
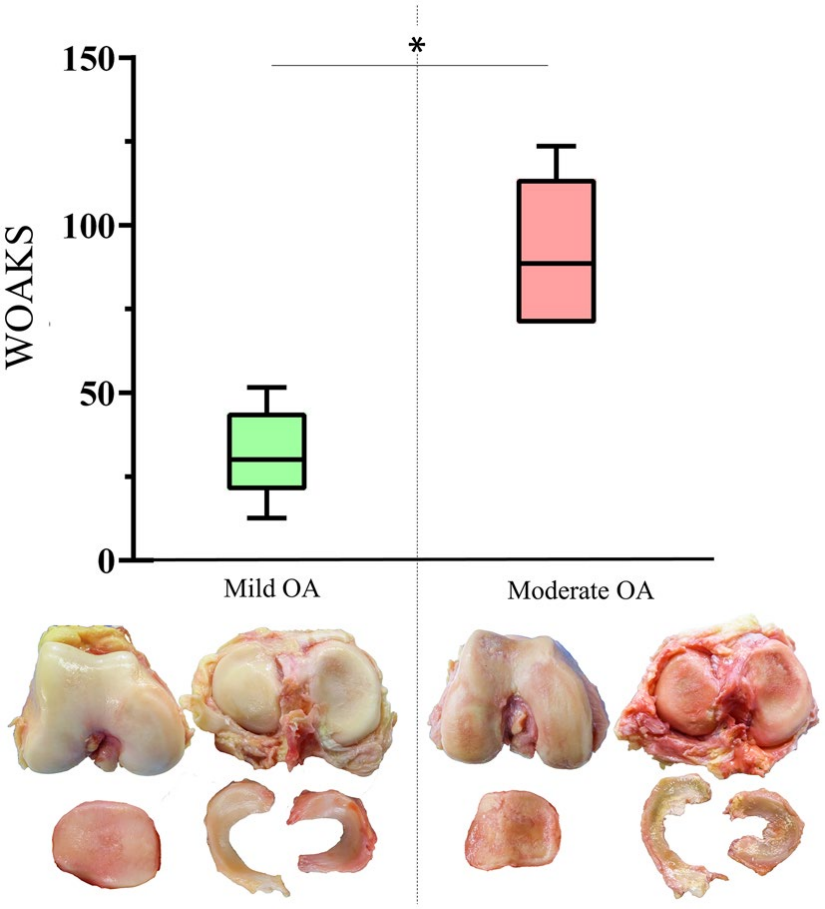
Results of Whole-organ arthroscopic knee score (WOAKS); Box-plots (min., mean, max., 25% and 75% percentiles) of the mild and severe OA knee joints according to the International Cartilage Research Society [21] and the Pauli degeneration score [28] with according, representative images of the cartilage surfaces and the menisci for both degeneration groups. Unpaired t-tests; *n* = 6; *p ≤ 0.05

The WOAKS of the mild OA joints ranged between 12 and 51, while that of the moderate OA joints was in the range of 70–123 (Table 1), where the mean WOAKS of the moderate OA joints was significantly higher (*p* < 0.0001) than that of the mild OA joints (Fig. 2).

**Table 1:** Overview of the WOAKS of the mild and moderate OA knee joints

Joint structure	Mild OA *n* = 6 specimens						Moderate OA *n* = 6 specimens					
	1	2	3	4	5	6	1	2	3	4	5	6
Femur (max 84)	3	18	7	2	3	3	27	19	28	21	27	25
Tibia (max 72)	6	12	6	6	13	6	9	18	20	14	7	9
Patella (max 36)	12	14	8	4	8	10	15	25	19	14	9	9
Menisci (max 72)	3	7	2	0	17	16	19	42	56	61	29	27
Total WOAKS (max 264)	24	51	23	12	41	32	70	104	123	110	72	70

The maximum score in the parentheses for each joint structure represents the number of evaluated locations multiplied by the maximum degeneration grade

### Passive pendulum tests

**Fig. 3:**
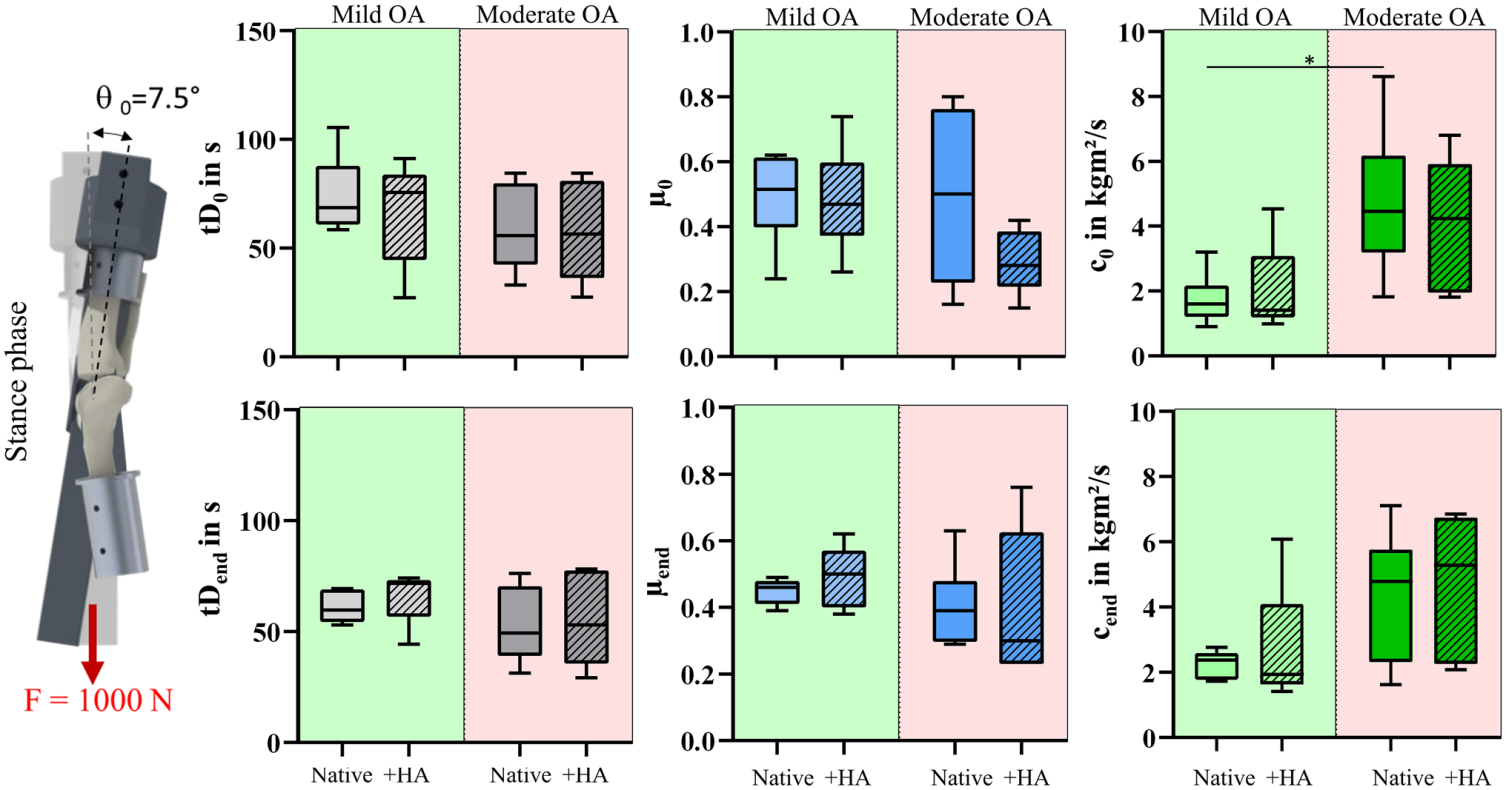
Results of the pendulum tests under stance phase conditions; Boxplots (min., max., median, 25% and 75% percentiles) of the damping time (tD_0/end_ in s), friction coefficient (µ_0/end_) and viscous damping coefficient (*c*_0__/end_ in kg m^2^/s) of the mild (green) and moderate OA knee joints (red) in the native condition (Native) and after hyaluronic acid supplementation (+ HA) determined under stance phase loading conditions. Wilcoxon and Mann–Whitney test; *n* = 6

**Fig. 4:**
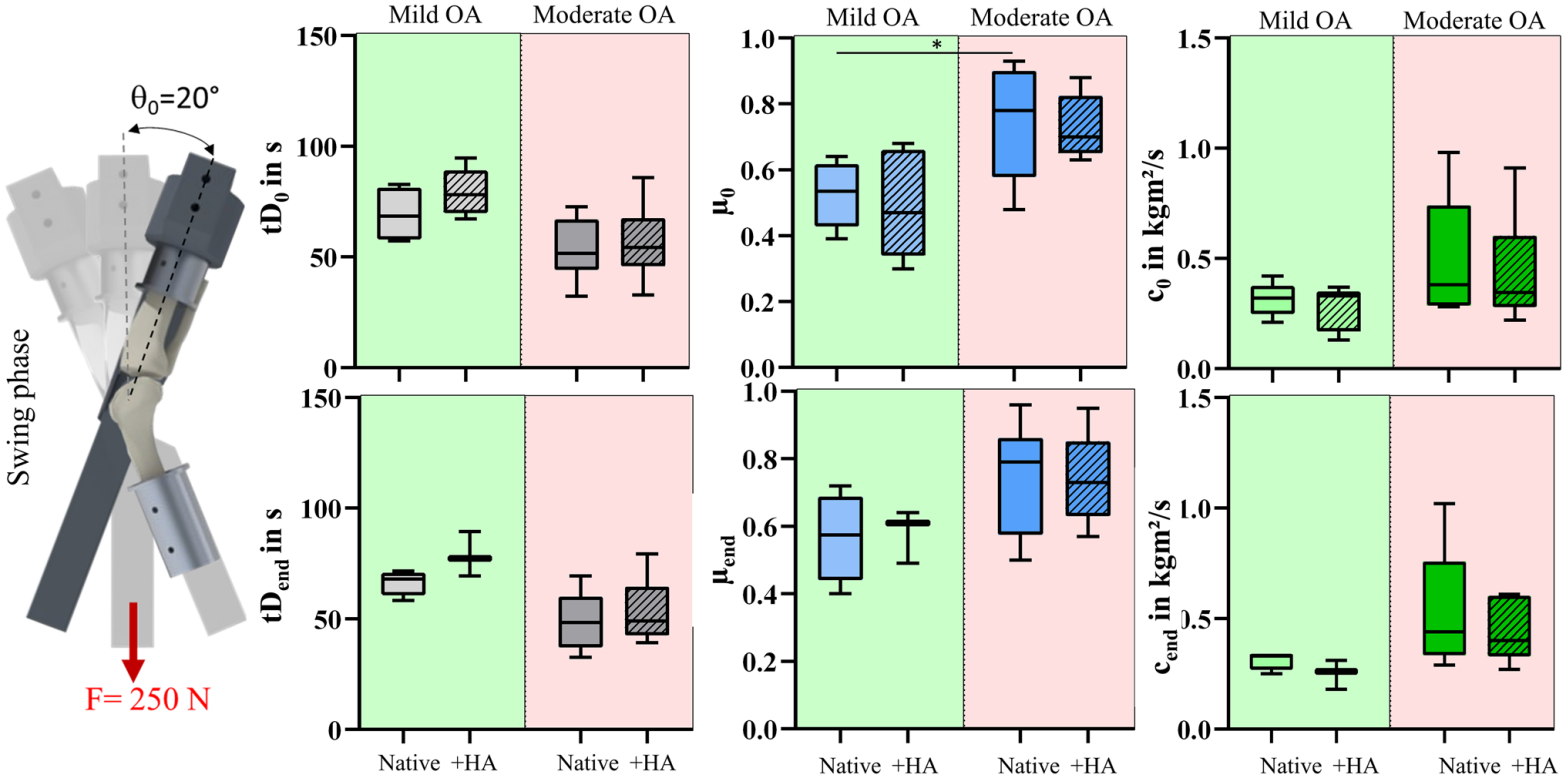
Results of the pendulum tests under swing phase conditions; Boxplots (min., max., median, 25% and 75% percentiles) of the damping time (tD_0/end_ in s), friction coefficient (*µ*_0__/end_) and viscous damping coefficient (*c*_0__/end_in kg m^2^/s) of the mild (green) and moderate OA knee joints (red) in the native condition (Native) and after hyaluronic acid supplementation (+ HA) determined under swing phase loading conditions. Wilcoxon and Mann–Whitney test; *n* = 6

No differences in the tD_0_ between the mild and moderate OA joints were observed under both, stance and swing phase conditions (Figs. 3, 4, Table 2). For the tD_end_, no differences were found between the mild and moderate OA joints, neither under stance nor under swing phase conditions (Figs. 3, 4, Table 2). Under swing phase conditions, the mild OA joints indicated significantly lower µ_0_ values (*p* < 0.05) than the moderate OA joints. Under stance phase conditions, *c*_0_ values were significantly higher in the moderate OA group (*p* = 0.01). No degeneration-related statistical differences were found for *µ*_end_ or *c*_end_. HA supplementation did not significantly alter the tD_0_, *µ*_0_ or *c*_0_ in any of the investigated conditions, which was also true for tD_end_, *µ*_end_ or *c*_end._

**Table 2:** Median [min, max] values of the passive pendulum test results for the mild and moderate OA joints under stance and swing phase conditions

	Mild OA		Moderate OA	
	Native	+ HA	Native	+ HA
*Stance phase*				
tD_0_ in s	69 [58, 105]	76 [27, 91]	56 [33, 84]	56 [27, 84]
*µ*_0_	0.52 [0.24, 0.62]	0.47 [0.26, 0.74]	0.50 [0.16, 0.80]	0.28 [0.15, 0.42]
*c*_0_ kg m^2^/s	1.60 [0.90, 3.20]	1.41 [1.00, 4.54]	4.45 [1.82, 8.62]	4.24 [1.81, 6.80]
tD_end_ in s	60 [53, 69]	72 [44, 74]	49 [31, 76]	53 [29, 78]
*µ*_end_	0.46 [0.39, 0.49]	0.50 [0.38, 0.62]	0.39 [0.29, 0.63]	0.30 [0.23, 0.76]
*c*_end_ kg m^2^/s	2.38 [1.73, 2.77]	1.94 [1.41, 6.08]	4.79 [1.63, 7.11]	5.29 [2.09, 6.85]
*Swing phase*				
tD_0_ in s	68 [57, 83]	78 [67, 95]	52 [32, 73]	54 [33, 86]
*µ*_0_	0.54 [0.39, 0.64]	0.47 [0.30, 0.68]	0.78 [0.48, 0.93]	0.70 [0.63, 0.88]
*c*_0_ kg m^2^/s	0.32 [0.21, 0.42]	0.33 [0.13, 0.37]	0.38 [0.28, 0.98]	0.35 [0.22, 0.91]
tD_end_ in s	68 [58, 72]	77 [69, 89]	48 [33, 69]	49 [39, 79]
*µ*_end_	0.58 [0.40, 0.72]	0.61 [0.49, 0.64]	0.79 [0.50, 0.96]	0.73 [0.57, 0.95]
*c*_end_ kg m^2^/s	0.33 [0.25, 0.34]	0.26 [0.18, 0.31]	0.44 [0.29, 1.02]	0.40 [0.27, 0.61]

### Pin-on-plate tests

**Fig. 5:**
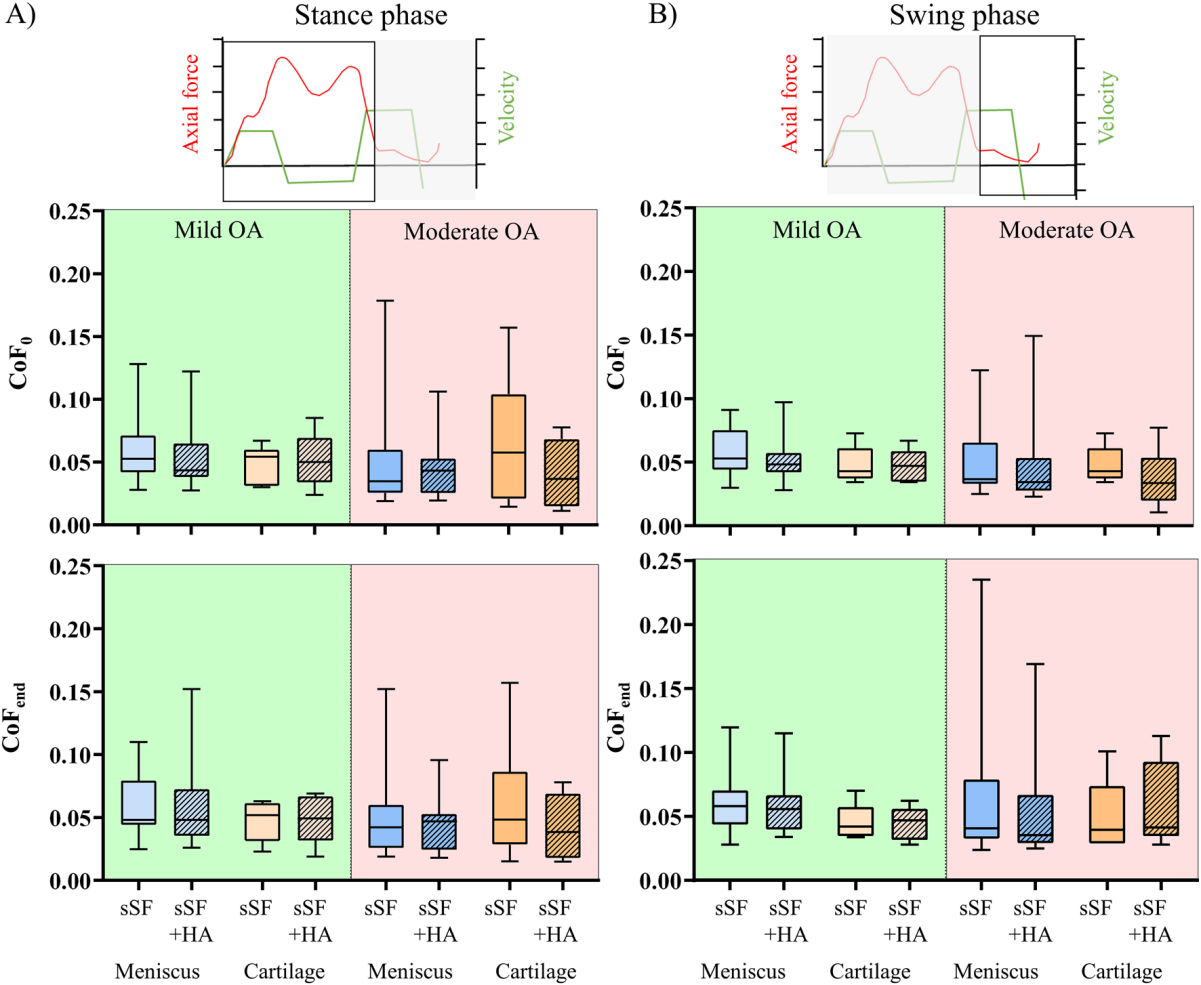
Results of the pin-on-plate tests; Boxplots (min., max., median, 25% and 75% percentiles) of the coefficient of friction (CoF) under stance (**A**) and swing phase (**B**) conditions. Results of the tribosystem Meniscus (*n* = 18) and Cartilage (*n* = 6) are shown for the samples with mild OA (green) and moderate OA (red), lubricated with synthetic synovial fluid (sSF) or with sSF and hyaluronic acid (HA) (sSF + HA). Wilcoxon and Mann–Whitney test

The CoF_0_ was not different for any of the tested tissue pairings, neither under stance nor under swing phase conditions (Fig. 5, Table 3). The lubricant comparisons between sSF and sSF + HA did not show any CoF_0_ differences, neither for the tribosystem Meniscus nor the tribosystem Cartilage (*p* > 0.05). For the CoF_end_, no differences were found between the mild and moderate OA joints, neither under stance nor under swing phase conditions (Fig. 5). Neither the tribosystem Meniscus nor the tribosystem Cartilage displayed any CoF_end_ differences for the lubricant comparisons between sSF and sSF + HA.

**Table 3:** Results of the pin-on-plate tests

	Mild OA		Moderate OA	
	sSF	sSF + HA	sSF	sSF + HA
*Stance phase*				
Meniscus				
CoF_0_	0.05 [0.03, 0.13]	0.04 [0.03, 0.12]	0.03 [0.02, 0.18]	0.04 [0.02, 0.11]
CoF_end_	0,05 [0.02, 0.11]	0.05 [0.03, 0.15]	0.04 [0.02, 0.10]	0.05 [0.02, 0.10]
Cartilage				
CoF_0_	0.05 [0.03, 0.07]	0.05 [0.02, 0.09]	0.06 [0.01, 0.16]	0.04 [0.01, 0.08]
CoF_end_	0.05 [0.02, 0.06]	0.05 [0.02, 0.07]	0.05 [0.02, 0.16]	0.04 [0.02, 0.08]
*Swing phase*				
Meniscus				
CoF_0_	0.05 [0.03, 0.09]	0.05 [0.03, 0.09]	0.04 [0.03, 0.12]	0.03 [0.02, 0.15]
CoF_end_	0.06 [0.03, 0.12]	0.06 [0.03, 0.12]	0.04 [0.02, 0.24]	0.04 [0.03, 0.17]
Cartilage				
CoF_0_	0.04 [0.03, 0.07]	0.05 [0.03, 0.07]	0.04 [0.03, 0.07]	0.03 [0.01, 0.08]
CoF_end_	0.04 [0.03, 0.07]	0.05 [0.03, 0.06]	0.04 [0.03, 0.10]	0.04 [0.03, 0.11]

Median [min, max] coefficient of friction (CoF) values of the tribosystem meniscus and cartilage for the samples with mild OA and moderate OA, lubricated with synthetic synovial fluid (sSF) or with sSF and hyaluronic acid (HA) (sSF + HA)

## Discussion

The most important finding of this study was that neither the age-related degeneration state of the cadaveric human knee joints nor the HA supplementation resulted in an altered friction, thus, disproving our hypotheses.

Joint lubrication involves three lubrication regimes: fluid film lubrication by the SF, boundary lubrication by molecules on the tissues surfaces and the SF and by pressurization of the interstitial fluid (IFP) in the cartilage matrix [36, 37]. Boundary lubrication is believed to be the predominant lubrication regime during the stance phase of gait, which is associated with high loading and low velocity [38]. During the swing phase, which is associated with low loading and high velocity, the friction is mainly guided through fluid-related lubrication mechanisms [38]. These lubrication regimes do not act concurrently but rather synergistically, whereby the transition between different lubrication regimes during gait lead to a so-called mixed lubrication regime [39]. In addition to the varying loading conditions during gait, the lubrication regimes are determined by the tissue structure and the composition of the SF [40]. Therefore, from the tribological aspect, it is reasonable to assume that degeneration-related changes impair joint lubrication, which can cause increased friction [3, 16, 38, 41–43]. However, the results from our complementary study indicated no impact of age-related degeneration on both, whole cadaveric knee joints and the subsequently extracted samples from these joints. Caligaris et al. found similar results for osteoarthritic human cartilage (Grades 1–3) tested in a pin-on-plate setup under quasi-static loading conditions [2]. They assumed that the friction-minimizing IFP appears to sustain maintained, despite the ongoing tissue composition changes and related surface texture alterations during the degeneration progression and the associated surface roughening [2]. The findings of the present study extend the results from Caligaris et al. by investigating gait-relevant loading conditions which are known to affect the friction behavior of articular cartilage [41, 44]. Therefore, based on our results, it can be concluded that the IFP can be maintained under the swing and stance phases of gait, suggesting that the knee joint appears to be able to compensate friction-related degenerative changes by adaptation of the lubrication [2].

One therapeutic mechanism of HA injections is the improvement of joint lubrication, thus, being able to restore the native low-friction environment in the knee joint [8, 16]. Our results showed no friction changes after HA supplementation, which is in contrast to earlier in vitro HA friction studies [14–16]. Numerous differences to existing studies might explain this. First, the lubrication regimes might differ. Previous studies on whole animal joints or extracted samples explicitly simulated either boundary or fluid lubrication [45–47]. The gait-like loading applied in the present study is likely to cause an interplay of different lubrication regimes. The literature suggests that the effect of HA on friction depends on the applied loads and thus the lubrication regime. Bell et al. analyzed the impact of mechanical surface roughening of bovine cartilage samples on boundary and fluid-guided lubrication. They found evidence that HA significantly reduces joint friction under quasi-static loading, but not under dynamic conditions [14]. Forsey et al. investigated the effect of HA on severely degenerated human OA cartilage samples (Grades 3–4) in the boundary lubrication regime and found that HA significantly reduced friction compared to PBS [16]. Second, different tissue source and OA phenotypes may contribute to the controversial outcome. In the present study, human knee joints with naturally progressing OA were investigated. Whole joint pendulum studies were previously performed on porcine hip [13] and temporomandibular joints [11] and bovine knee joints [47]. There is evidence that age-dependent OA changes in human knees are different from those obtained in OA-induced animal models. Moreover, in the animal studies the mechanical OA-like changes were simulated by scouring with gauze [11, 13] or sandpaper [13]. Such an in vitro mechanical surface deterioration is very likely not the same as compared with the in vivo OA degeneration progression, which affects both the structure and composition of the involved tissues. Obara et al. [48] considered the natural progression of the disease in their rabbit OA model. Degeneration was induced in vivo by papain injections or with the transection of the anterior cruciate ligament. Similar to the present study design, they applied the HA viscosupplementation after the animals were euthanized and tested the effect by means of pendulum friction tests. They found significantly reduced friction only in joints with slight surface irregularities, but not in those with advanced cartilage fibrillation, as it can be observed in higher OA grades. It was suggested that in vitro supplemented HA is unable to improve lubrication once the degeneration manifests itself in macroscopic tissue changes [48]. Deterioration of the cartilage surface may change the concentration of boundary macromolecules like lubricin or phospholipids that interact with HA to promote low friction [3, 49]. This in vitro study is not without limitations. First, passive pendulum tests do not account for the contribution of muscle forces to joint loading [22, 32], thus, potentially leading to lower joint contact pressures*,* which might also affect the prevalent lubrication regime [38]. To mitigate this limitation, the axial loads applied in the present study were directly derived from in vivo measurements of instrumented prostheses [24]. Second, instead of using patient-specific SF for the pin-on-plate tests, we used sSF as lubricant. However, the MW of the sSF was comparable to those of diseased joints [34], thus, qualifying it to be an adequate control lubricant because the relubrication effect of HA supplementation is known to be mainly attributed to added high MW HA in a low MW HA environment [1]. Third, the extracted samples of the pin-on-plate tests were used after performing the pendulum tests with associated HA viscosupplementation. Corvelli et al. showed in their in vitro study on bovine cartilage tissue that supplemented HA does not penetrate the cartilage but rather remains on the surface [15]. Before testing, we rinsed the extracted samples in PBS to minimize the lubrication effects of the before supplemented HA. Fourth, the supplemented HA (Hylan G-F 20) indicated a MW of 6000 kDa, while previous in vitro studies which used a HA viscosupplementation with only 1000–2350 kDa MW found a friction-reducing effect [13, 15]. However, both, Bonnevie et al. and Rebenda et al. did not find a dependence of HA MW on cartilage friction [9, 50]. Therefore, we assume that discrepancies between our results and existing studies are not associated with the HA MW. Moreover, it should be noted that the OA is a very multifactorial disease with a wide spectrum. Although, we assigned the tested specimens into a mild and moderate OA group according to the donor age and verified by WOAKS score this might limits the interpretation of our findings.

Although we did not find an initial effect of HA supplementation on human knee joint friction in vitro, there is evidence that in vivo supplemented HA contributed to the maintenance of a low-friction environment in a leporine OA model over a period of 9 weeks [12]. In-vivo, supplemented HA can remain in the joint for few days [8], which was postulated to have a disease-modifying effect [8]. By covering the cartilage surface and filling the space between the collagen fibres, a loss of proteoglycans is inhibited [51]. Moreover, it was shown on fibroblasts that endogenous HA production is stimulated by exogenous HA supplementation [52] and that HA has an anti-inflammatory effect [53]. Thus, a synergy of different therapeutic mechanisms might be responsible for the effective clinical outcome in terms of pain relief and improving knee function [8, 54]. However, in the present in vitro study on cadaveric human knee joints it was not possible to investigate the biological effects of HA. Unfortunately, investigations of long term in vivo effects of HA supplementation on joint friction in the human knee are challenging because there is currently no method to determine in vivo joint friction. In a recent study, Kupratis et al. showed that cartilage lubricity correlates with the biomechanical properties of the involved joint tissues [55] and, moreover, it was also demonstrated that biomechanical properties can be predicted with quantitative magnetic resonance imaging [56, 57]. Therefore, future tribological research on the correlations between friction and in vivo assessed biomechanical properties might allow for long term in vivo friction monitoring with the aim to identify sufficient viscosupplementations to reduce the rate of lubrication-related OA progression [55].

## Conclusion

The findings of this two-part tribological in-vitro study extend the literature on the friction properties human knee joints with different degrees of degeneration. The results indicated that progressing degeneration of knee joint tissue is not characterized by increased friction. Moreover, HA supplementation did not initially decrease the in vitro friction, neither on the joint nor the tissue level. This might indicate that the therapeutic mechanisms of HA do not involve the initial improvement of friction.

## Data Availability

The data that support the findings of this study are available on request from the authors.

## Abbreviations

OA: Osteoarthritis
HA: Hyaluronic acid
MW: Molecular weight
CoF: Coefficient of friction
WOAKS: Whole-organ arthroscopic knee score
sSF: Synthetic synovial fluid
IFP: Interstitial fluid pressurization

## References

[1] Gilat R, Haunschild ED, Knapik DM, Evuarherhe A, Parvaresh KC, Cole BJ (2021). Hyaluronic acid and platelet-rich plasma for the management of knee osteoarthritis. Int Orthop.

[2] Caligaris M, Canal CE, Ahmad CS, Gardner TR, Ateshian GA (2009). Investigation of the frictional response of osteoarthritic human tibiofemoral joints and the potential beneficial tribological effect of healthy synovial fluid. Osteoarthr Cartil.

[3] Desrochers J, Amrein MW, Matyas JR (2013). Microscale surface friction of articular cartilage in early osteoarthritis. J Mech Behav Biomed Mater.

[4] Neu CP, Reddi AH, Komvopoulos K, Schmid TM, Di Cesare PE (2010). Increased friction coefficient and superficial zone protein expression in patients with advanced osteoarthritis. Arthritis Rheum.

[5] Cooper C, Rannou F, Richette P, Bruyère O, Al-Daghri N, Altman RD, Brandi ML, Collaud Basset S, Herrero-Beaumont G, Migliore A, Pavelka K, Uebelhart D, Reginster JY (2017). Use of intraarticular hyaluronic acid in the management of knee osteoarthritis in clinical practice. Arthritis Care Res (Hoboken).

[6] Petrella RJ, Cogliano A, Decaria J (2008). Combining two hyaluronic acids in osteoarthritis of the knee: a randomized, double-blind, placebo-controlled trial. Clin Rheumatol.

[7] Nguyen C, Lefèvre-Colau M-M, Poiraudeau S, Rannou F (2016). Evidence and recommendations for use of intra-articular injections for knee osteoarthritis. Ann Phys Rehabil Med.

[8] Altman RD, Manjoo A, Fierlinger A, Niazi F, Nicholls M (2015). The mechanism of action for hyaluronic acid treatment in the osteoarthritic knee: a systematic review. BMC Musculoskelet Disord.

[9] Bonnevie ED, Galesso D, Secchieri C, Bonassar LJ (2019). Frictional characterization of injectable hyaluronic acids is more predictive of clinical outcomes than traditional rheological or viscoelastic characterization. PLoS ONE.

[10] Chavda S, Rabbani SA, Wadhwa T (2022). Role and effectiveness of intra-articular injection of hyaluronic acid in the treatment of knee osteoarthritis: a systematic review. Cureus.

[11] Kawai N, Tanaka E, Takata T, Miyauchi M, Tanaka M, Todoh M, van Eijden T, Tanne K (2004). Influence of additive hyaluronic acid on the lubricating ability in the temporomandibular joint. J Biomed Mater Res A.

[12] Kawano T, Miura H, Mawatari T, Moro-Oka T, Nakanishi Y, Higaki H, Iwamoto Y (2003). Mechanical effects of the intraarticular administration of high molecular weight hyaluronic acid plus phospholipid on synovial joint lubrication and prevention of articular cartilage degeneration in experimental osteoarthritis. Arthritis Rheum.

[13] Mori S, Naito M, Moriyama S (2002). Highly viscous sodium hyaluronate and joint lubrication. Int Orthop.

[14] Bell CJ, Ingham E, Fisher J (2006). Influence of hyaluronic acid on the time-dependent friction response of articular cartilage under different conditions. Proc Inst Mech Eng H.

[15] Corvelli M, Che B, Saeui C, Singh A, Elisseeff J (2015). Biodynamic performance of hyaluronic acid versus synovial fluid of the knee in osteoarthritis. Methods.

[16] Forsey RW, Fisher J, Thompson J, Stone MH, Bell C, Ingham E (2006). The effect of hyaluronic acid and phospholipid based lubricants on friction within a human cartilage damage model. Biomaterials.

[17] Mederake M, Trappe D, Jacob C, Hofmann UK, Schüll D, Dalheimer P, Exner L, Walter C (2022). Influence of hyaluronic acid on intra-articular friction—a biomechanical study in whole animal joints. BMC Musculoskelet Disord.

[18] Martin-Alarcon L, Schmidt TA (2016). Rheological effects of macromolecular interactions in synovial fluid. Biorheology.

[19] Warnecke D, Meßemer M, de Roy L, Stein S, Gentilini C, Walker R, Skaer N, Ignatius A, Dürselen L (2019). Articular cartilage and meniscus reveal higher friction in swing phase than in stance phase under dynamic gait conditions. Sci Rep.

[20] Li Y, Yuan Z, Yang H, Zhong H, Peng W, Xie R (2021). Recent advances in understanding the role of cartilage lubrication in osteoarthritis. Molecules.

[21] Kleemann RU, Krocker D, Cedraro A, Tuischer J, Duda GN (2005). Altered cartilage mechanics and histology in knee osteoarthritis: relation to clinical assessment (ICRS Grade). Osteoarthr Cartil.

[22] de Roy L, Warnecke D, Hacker SP, Simon U, Dürselen L, Ignatius A, Seitz AM (2021). Meniscus injury and its surgical treatment does not increase initial whole knee joint friction. Front Bioeng Biotechnol.

[23] Bonnefoy-Mazure A, Armand S (2015) Normal gait. 199–214

[24] ISO 14243-1 (2009) Implants for surgery–wear of total knee joint prostheses—part 1: loading and displacement parameters for wear-testing machines with load control and corresponding environmental conditions for test

[25] Shane Anderson A, Loeser RF (2010). Why is osteoarthritis an age-related disease?. Best Pract Res Clin Rheumatol.

[26] Spahn G, Mückley T, Klinger HM, Hofmann GO (2008). Whole-organ arthroscopic knee score (WOAKS). BMC Musculoskelet Disord.

[27] Robert H, Lambotte JC, Flicoteaux R (2011). Arthroscopic measurement of cartilage lesions of the knee condyle: principles and experimental validation of a new method. Cartilage.

[28] Pauli C, Grogan SP, Patil S, Otsuki S, Hasegawa A, Koziol J, Lotz MK, D'Lima DD (2011). Macroscopic and histopathologic analysis of human knee menisci in aging and osteoarthritis. Osteoarthr Cartil.

[29] Freeman MAR, Pinskerova V (2005). The movement of the normal tibio-femoral joint. J Biomech.

[30] Stein S, Höse S, Warnecke D, Gentilini C, Skaer N, Walker R, Kessler O, Ignatius A, Dürselen L (2019). Meniscal replacement with a silk fibroin scaffold reduces contact stresses in the human knee. J Orthop Res.

[31] Radecki J, Kim S, Vad D (2009). Synvisc-One™ for the treatment of knee osteoarthritis. Int J Clin Rheumatol.

[32] Crisco J, Blume J, Teeple E, Fleming B, Jay G (2007). Assuming exponential decay by incorporating viscous damping improves the prediction of the coeffcient of friction in pendulum tests of whole articular joints. Proc Inst Mech Eng H J Eng Med.

[33] Warnecke D, Schild NB, Klose S, Joos H, Brenner RE, Kessler O, Skaer N, Walker R, Freutel M, Ignatius A, Dürselen L (2017). Friction properties of a new silk fibroin scaffold for meniscal replacement. Tribol Int.

[34] Bortel E, Charbonnier B, Heuberger R (2015). Development of a synthetic synovial fluid for tribological testing. Lubricants.

[35] Heilmann HH, Lindenhayn K, Walther HU (1996). Das Synovia-Volumen gesunder und arthrotischer menschlicher Kniegelenke. Zeitschrift Fur Orthopadie Und Unfallchirurgie - Z ORTHOP UNFALLCHIR.

[36] Ateshian GA (2009). The role of interstitial fluid pressurization in articular cartilage lubrication. J Biomech.

[37] Lin W, Klein J (2021). Recent progress in cartilage lubrication. Adv Mater.

[38] Neu CP, Komvopoulos K, Reddi AH (2008). The interface of functional biotribology and regenerative medicine in synovial joints. Tissue Eng B Rev.

[39] Murakami T, Yarimitsu S, Sakai N, Nakashima K, Yamaguchi T, Sawae Y (2017). Importance of adaptive multimode lubrication mechanism in natural synovial joints. Tribol Int.

[40] Link JM, Salinas EY, Hu JC, Athanasiou KA (2020). The tribology of cartilage: Mechanisms, experimental techniques, and relevance to translational tissue engineering. Clin Biomech (Bristol, Avon).

[41] Furmann D, Nečas D, Rebenda D, Čípek P, Vrbka M, Křupka I, Hartl M (2020). The effect of synovial fluid composition, speed and load on frictional behaviour of articular cartilage. Materials (Basel).

[42] Lee SS, Duong CT, Park SH, Cho Y, Park S, Park S (2013). Frictional response of normal and osteoarthritic articular cartilage in human femoral head. Proc Inst Mech Eng H.

[43] Peck J, Slovek A, Miro P, Vij N, Traube B, Lee C, Berger AA, Kassem H, Kaye AD, Sherman WF, Abd-Elsayed A (2021). A comprehensive review of viscosupplementation in osteoarthritis of the knee. Orthop Rev (Pavia).

[44] Chatterjee A, Dubey DK, Sinha SK (2021). Effect of loading on the adhesion and frictional characteristics of top layer articular cartilage nanoscale contact: a molecular dynamics study. Langmuir.

[45] Teeple E, Fleming BC, Mechrefe AP, Crisco JJ, Brady MF, Jay GD (2007). Frictional properties of Hartley guinea pig knees with and without proteolytic disruption of the articular surfaces. Osteoarthr Cartil.

[46] Akelman MR, Teeple E, Machan JT, Crisco JJ, Jay GD, Fleming BC (2013). Pendulum mass affects the measurement of articular friction coefficient. J Biomech.

[47] Mabuchi K, Obara T, Ikegami K, Yamaguchi T, Kanayama T (1999). Molecular weight independence of the effect of additive hyaluronic acid on the lubricating characteristics in synovial joints with experimental deterioration. Clin Biomech (Bristol, Avon).

[48] Obara T, Mabuchi K, Iso T, Yamaguchi T (1997). Increased friction of animal joints by experimental degeneration and recovery by addition of hyaluronic acid. Clin Biomech.

[49] Bonnevie ED, Bonassar LJ (2020). A century of cartilage tribology research is informing lubrication therapies. J Biomech Eng.

[50] Rebenda D, Ranuša M, Čípek P, Toropitsyn E, Vrbka M (2023). In situ observation of hyaluronan molecular weight effectiveness within articular cartilage lubrication. Lubricants.

[51] Balazs E, Helfet AJ (1982). The pysical properties of synovial fluid and the specific role of hyaluronic acid. Disorders of the Knee.

[52] Smith MM, Ghosh P (1987). The synthesis of hyaluronic acid by human synovial fibroblasts is influenced by the nature of the hyaluronate in the extracellular environment. Rheumatol Int.

[53] Goto M, Hanyu T, Yoshio T, Matsuno H, Shimizu M, Murata N, Shiozawa S, Matsubara T, Yamana S, Matsuda T (2001). Intra-articular injection of hyaluronate (SI-6601D) improves joint pain and synovial fluid prostaglandin E2 levels in rheumatoid arthritis: a multicenter clinical trial. Clin Exp Rheumatol.

[54] Wang M, Liu C, Thormann E, Dėdinaitė A (2013). Hyaluronan and phospholipid association in biolubrication. Biomacromol.

[55] Kupratis ME, Gure AE, Benson JM, Ortved KF, Burris DL, Price C (2022). Comparative tribology II-measurable biphasic tissue properties have predictable impacts on cartilage rehydration and lubricity. Acta Biomater.

[56] Wheaton AJ, Dodge GR, Elliott DM, Nicoll SB, Reddy R (2005). Quantification of cartilage biomechanical and biochemical properties via T1rho magnetic resonance imaging. Magn Reson Med.

[57] Nieminen MT, Töyräs J, Laasanen MS, Silvennoinen J, Helminen HJ, Jurvelin JS (2004). Prediction of biomechanical properties of articular cartilage with quantitative magnetic resonance imaging. J Biomech.

